# INTEREST GROUP SESSION—NUTRITION: IMPLEMENTATION OF NUTRITION, BODY COMPOSITION, AND FUNCTIONAL ASSESSMENT IN VULNERABLE OLDER ADULTS

**DOI:** 10.1093/geroni/igz038.1440

**Published:** 2019-11-08

**Authors:** Rose Ann DiMaria-Ghalili, Julie Locher

**Affiliations:** 1 Drexel University, Philadelphia, Pennsylvania, United States; 2 University of Alabama at Birmingham, Birmingham, Alabama, United States

## Abstract

This symposium will highlight the implementation of nutrition, body composition and functional assessment in vulnerable older adults. Understanding the barriers and enablers of implementing traditional nutrition screening and assessment tools, nutrition focused physical examination, body composition analysis, functional assessment and biomarkers can aid researchers in the design of pragmatic nutrition studies in vulnerable older adults. DiMaria-Ghalili will provide an introduction to overall nutrition assessment in community-dwelling older adults and present lessons learned in regards to nutrition assessment protocols (Mini-Nutritional Assessment, hand-grip strength, and inflammatory biomarkers) from an ongoing clinical trial. Starr will discuss the issues surrounding nutrition screening and assessment in high-risk older adults undergoing abdominal surgery. A comparison of data on different nutrition screening and assessment tools (NRS-2002; PG-SGA, Nutrition Focused Physical Examination) in older adults undergoing abdominal surgery will be presented. Batsis will discuss the controversies and challenges in body composition assessment focusing on sarcopenic obesity. Data highlighting the different diagnostic criteria of sarcopenic obesity and diagnostic accuracy will be presented. The link between overall nutrition status and physical function on health outcomes is emerging in older adults. Bales will discuss the types of physical function assessment tools used in studies of older adults and discuss her findings on the impact of higher protein intake on functional outcomes. The symposium will conclude with a discussion led by Locher on practical implications of integrating best assessment techniques into clinical practice.

